# Evaluation of Polyethylene Glycol Diacrylate-Polycaprolactone Scaffolds for Tissue Engineering Applications

**DOI:** 10.3390/jfb8030039

**Published:** 2017-09-05

**Authors:** Hari Kotturi, Alaeddin Abuabed, Haris Zafar, Elaine Sawyer, Bipin Pallipparambil, Harsha Jamadagni, Morshed Khandaker

**Affiliations:** 1 Department of Biology, University of Central Oklahoma, Edmond, OK 73034, USA; HKotturi@uco.edu (H.K.); hzafar@uco.edu (H.Z.); esawyer1@uco.edu (E.S.); 2 Department of Engineering & Physics, University of Central Oklahoma, Edmond, OK 73034, USA; Aabuabed@uco.edu (A.A.); bpallipparambilvarg@uco.edu (B.P.); hjamadagni@uco.edu (H.J.)

**Keywords:** polyethylene glycol diacrylate, polycaprolactone, scaffold, electrospun, nanofiber

## Abstract

Polyethylene Glycol Diacrylate (PEGDA) tissue scaffolds having a thickness higher than 1 mm and without the presence of nutrient conduit networks were shown to have limited applications in tissue engineering due to the inability of cells to adhere and migrate within the scaffold. The PEGDA scaffold has been coated with polycaprolactone (PCL) electrospun nanofiber (ENF) membrane on both sides to overcome these limitations, thereby creating a functional PEGDA-PCL scaffold. This study examined the physical, mechanical, and biological properties of the PEGDA and PEGDA-PCL scaffolds to determine the effect of PCL coating on PEGDA. The physical characterization of PEGDA-PCL samples demonstrated the effectiveness of combining PCL with a PEGDA scaffold to expand its applications in tissue engineering. This study also found a significant improvement of elasticity of PEGDA due to the addition of PCL layers. This study shows that PEGDA-PCL scaffolds absorb nutrients with time and can provide an ideal environment for the survival of cells. Furthermore, cell viability tests indicate that the cell adhered, proliferated, and migrated in the PEGDA-PCL scaffold. Therefore, PCL ENF coating has a positive influence on PEGDA scaffold.

## 1. Introduction

Tissue engineering (TE) holds great promise for the cultivation of patient-specific tissues for restoring organ functions and/or curing various diseases [1,2,3]. TE techniques involve seeding or implantation of cells into scaffolds that are biodegradable and capable of supporting three-dimensional (3D) cell growth. Photosensitive hydrogels, such as Polyethylene Glycol Diacrylate (PEGDA) are an important class of biomaterials with many TE applications [1,2,3].

Photolithography is a commonly used process in micro-fabrication to produce the desired scaffold with specific shape and size using a mold [4]. The ability to control the porosity of photosensitive hydrogel such as Polyethylene Glycol Diacrylate (PEGDA) to elicit altered cell behaviors, including cell adhesion, has raised heightened interest in the scaffold materials for various biomedical applications such as orthopedic repair and regeneration [5] and liver tissue engineering [6]. We have used PEGDA to generate 3D scaffolds in our research [7]. Several PEGDA hydrogel scaffolds have been developed for the reconstruction of injured hard and soft tissues, although the in vivo performances have not been reported yet [8,9]. The reason for using PEGDA in our research over other materials is that a thin layer of PEGDA membrane can be manufactured readily to allow for cell growth. In addition, any custom shape membrane can be fabricated using PEGDA using a 3D printed mold.

PEGDA scaffolds having a thickness higher than 1 mm were shown to have limited applications as a three-dimensional cell culture device due to the inability of cells to survive within the scaffolds [9]. Cells that are placed deep inside the PEGDA scaffold with a thickness higher than 1 mm die out due to the lack of having access to adequate nutrients. Lack of porosity in the PEGDA scaffold leads the cells to non-uniform tissue regeneration. PEGDA scaffolds need to be designed with intricate architecture, porosity, pore size and shape, and interconnectivity in order to provide the required structural strength, nutrient transport, and micro-environment for cell and tissue in-growth. There is a significant need for scientific research to investigate methods that can overcome the limitations exhibited by thick PEGDA for TE applications. Various PEGDA-based scaffolds were researched; however, none of them fulfill all the requirements for TE applications [10,11,12]. Overcoming the functional deficits of PEGDA for TE applications motivates this research.

Electrospinning is a process by which fibers with micro to nanometer diameters can be fabricated from an electrostatically driven jet of polymer solution. These fibers have a high surface area to volume ratio, which can have numerous industrial applications as barrier fabrics, wipes, medical, and pharmaceutical uses. In our early research, we have developed the electrospin process to produce polycaprolactone (PCL) electrospun nanofiber (ENF) membrane that has competing performances as a functional coating material. The thickness of our PCL ENF membrane is usually in the range of microns. In other research efforts, researchers have reported that the biological functions of ENF membrane depend upon the fiber material, fiber size, and thickness of the membrane [1]. There is no research to date conducted to determine the influence of ENF membrane on the mechanical and biological performances of the PEGDA scaffold. There is still a significant need for scientific research to overcome physical (porosity, water absorption), mechanical (stiffness, elasticity) and biological (cell adherence, proliferation, and migration) limitations of thick PEDGA hydrogel membranes for tissue engineering applications [13]. Therefore, the goal of this study focuses on the physical, mechanical and biological capabilities of PEDGA-PCL scaffold and evaluates the capabilities for tissue engineering applications.

## 2. Materials and Methods

### 2.1. Materials

Two solutions were combined to make the PEGDA hydrogel solution mix. The first solution consisted of the liquid Polyethylene Glycol Diacrylate (PEGDA), M_n_ = 700 (mol), diluted with liquid Dulbecco’s Phosphate Buffer Saline (PBS). The second solution consisted of a solute solid photo-initiator (PI) Alpha-alpha-dimethoxy-alpha-phenylacetophenone, M_w_ = 256.35 (g/mol) Sigma-Aldrich, St. Louis, MO, USA; which was dissolved in the liquid solvent 1-vinyl-2-pyrrolidone, M_w_ = 111.14 (g/mol). PCL beads (pellet size ~3 mm, average M_n_ 80,000) and acetone (laboratory reagent ≥99.5%) solvent were used to prepare the PCL solution.

### 2.2. Sample Preparation

#### 2.2.1. Sample Design

This study prepared two groups of cylindrical specimens: PEGDA and PEGDA-PCL. The specimen dimension closely depends on the silicone mold, which is 10 mm and 1.5 mm thickness. Each group of samples was prepared for six different experiments: SEM images of the surface and histological longitudinal section, Dulbecco’s modified Eagle’s medium (DMEM) absorption, compression, rheological, and cell viability tests.

#### 2.2.2. Sample Fabrication Process

The researchers in this study fabricated an electrospinning-UV polymerization system. The details of working principles of the system have been recently presented by Abuabed et al. [14]. The system can produce any dimension of cylindrical shape PEGDA-PCL scaffold. Specifically, this study fabricated 10 mm diameter and 1.5 mm thickness PEGDA-PCL scaffold. This dimension is selected due to the suitability of cell culturing for biocompatibility tests on each group of samples in 48-well plates.

**Figure 1:**
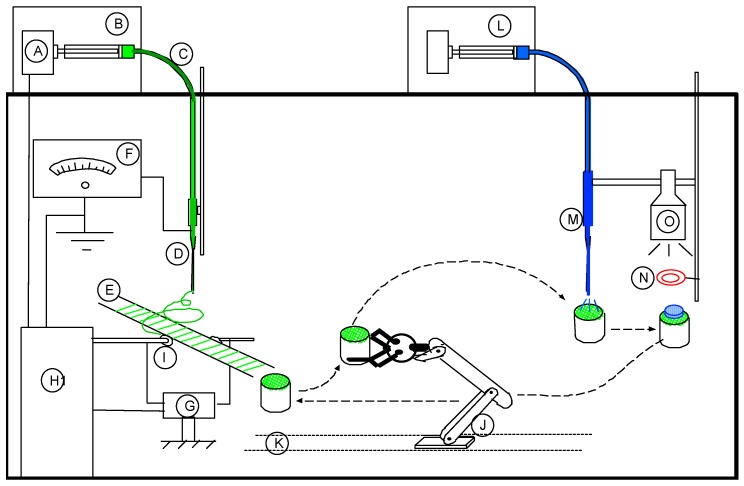
Schematic representation of a Polyethylene Glycol Diacrylate-Polycaprolactone (PEGDA-PCL scaffold [15]. (**A**) First syringe pump, (**B**) Glass syringe, (C) tube, (D) metallic needle, (E) two parallel wires, (F) high voltage source, (G) fixtures for holding fiber collector, (H) electrical signal controller, (I) connector to transfer electricity to wire, (J) robotic arm to transfer a mold from/to fiber collector to curing station, (K) mold for collection of fibers, (L) Second syringe pump, (M) sprayer, and (N) UV mask or mold to cure PEGDA.

Figure 1 illustrates functional elements of a notional combined ENF production-UV photopolymerization unit for automatic production of the 3D scaffold. The notional system combines an ENF production unit and UV polymerization unit and a robotic arm for fiber harvesting. Using a system with these functional elements, any number of PCL ENF and PEGDA membranes may be produced in any shape of 3D scaffolds. A substrate may be adapted to produce a 3D scaffold comprising at least two equal linear dimensions, or a circular shape. In this study, we have used circular shape collector as shown in Figure 1. Schematic representation of a PEGDA-PCL scaffold [15]. The process b-c-d can be repeated multiple times to create higher thickness scaffold. PCL pellets (7.69 wt %) were mixed with acetone in an ultrasonic mixer (Sonics & Materials, Inc., Newtown, CT, USA, model # Vibra-cell VCX 130). The sonication process was carried out at approximately 60 °C for 30 min. A syringe pump (Figure 1A) is used to feed PCL solution (2.5 milliliter) into a glass syringe (Figure 1B) and flow it through a tube (Figure 1C) to a metallic needle (Figure 1D). The metallic disc collectors (Figure 1E) are spun using speed controlled, direct current (DC) motors. The syringe needle (Figure 1D) is electrically excited by applying a high-voltage (15 Kilovolt) (Figure 1F) produced by the power supply. This electrically charged syringe needle for electrospinning synthetic polymer fiber streams is positioned above and substantially centered between the edges of metallic collectors. The distance between the needle and dual disc collectors was approximately 5 cm. The feeding rate of the PCL solution was adjusted to a rate of 0.025 mL/minute. This will realize an electrical potential difference between the needle tip and the disks, the positioning being adjustable by Z position control stage (Figure 1G). As a result, an electrostatic field is formed between the charged syringe needle and the edges of the rotating metallic disks. This enables capturing, depositing and aligning fiber between the rotating parallel collectors. A 25 × 25 × 25 mm square block with 10 mm diameter through hole is used to collect fiber. A “smart” robotic arm (Figure 1J) in a track (Figure 1K) is used to collect the twelve layers of aligned fibers from the parallel collector on the top of the acrylic substrate. A hollow silicone mold (inner diameter 10 mm and thickness 5 mm) is placed on top of an acrylic substrate such that the hole of the silicone mold aligned with the hole of the acrylic. The acrylic substrate feeds it to the curing station without manually intervening in this process.

The photoinitiator (Alpha-alpha-dimethoxy-alpha-phenylacetophenone) was mixed with the solvent (1-vinyl-2-pyrrolidone) to prepare photoinitiator mixture. PEGDA was mixed with PBS to prepare PEGDA mixture. The PEGDA and photoinitiator mixtures were mixed in the desired amount to prepare 0.2% and 0.6% of photoinitiator PEGDA. A second syringe pump system (Figure 1L) with spray/needle tip (Figure 1M) is used to deposit PEGDA solution (80 micro liter) on the top of a fiber matrix. The silicone mold (Figure 1N) is used to cure 1.5 mm thick PEGDA layer on the top of a fiber matrix using a UV light (Figure 1O).

### 2.3. Experiments

#### 2.3.1. SEM and Histological Examination

To observe the fiber embedding in PEGDA, the fiber layer surface of PEGDA-PCL scaffold was viewed under Hitachi^TM^ (Chiyoda, Tokyo, Japan) 3000 SEM. For PEGDA samples, SEM image was captured on the flat surface of PEGDA. To view the internal architecture of PEGDA and PEGDA-PCL scaffold, both groups of samples were embedded in paraffin. The paraffin embedded samples were cut longitudinally using a microtome. Multiple longitudinal micro sections were produced for SEM imaging.

#### 2.3.2. Mechanical Tests

Evex micro tensile/compression mechanical test system (Evex Analytical Instruments Inc., Princeton, NJ, USA) has been used for the compression test of both scaffolds. Each group of samples was mounted between the holders in the Evex test system. The sample was compressed to 80% of the gel height at a strain rate of 0.007 s^−1^ during the unconfined compression tests. The reason for using low strain rate during the mechanical test is because one of the intended purposes of our scaffold is liver tissue engineering. Studies on porcine liver tissues found that varying the strain rates from 0.003 to 0.6 s^−1^ did not have a significant effect on the stress–strain data for the compression and elongation experiments [16]. The load and the corresponding displacement of the scaffolds were directly recorded from EVEX machine software. The slopes of the curves were utilized to compare the difference in stiffness between the samples. Oscillation tests were performed on both group of samples using the Malvern CVO-100 (Malvern, UK) rheometer at 5% strain rate at frequency 1 Hz (a typical oscillation rate of tissue in a rodent under motion) [17]. Kerdok et al. studies [18] on in vivo and ex vivo perfused porcine liver model showed that the liver tissue behaved like an elastic spring at a frequency below 40 Hz. Therefore, viscous, elastic and complex moduli were found at a frequency of 1 Hz rather than a frequency sweep during the study.

#### 2.3.3. Bioactivity

##### A. Absorption Test

The in vitro bioactivity of the scaffolds was assessed by soaking each group of scaffolds in DMEM for 7, 14 and 21 days, respectively. The initial weight of the scaffold, W_0_, was measured. After each time period, the weight of the scaffold was measured, W_t_. The value of the rate of DMEM absorption in percentage was measured using the formula: (W_t_ − W_0_) × 100%/W_0_.

##### B. Cell Cultures

Human hepatocellular carcinoma cells (GS5 cells) were chosen as our study model cell type. There are two main reasons for selecting hepatocytes as cell prototype scaffolds in this study. Firstly, we have used hepatocyte cells in scaffolds with various compositions in our previous studies [19]. Secondly, multilayers of PEGDA-PCL structures can potentially be used in liver tissue engineering as in vitro model systems for fundamental and applied studies of liver function in healthy and diseased states. The cells used in this study were cultured and maintained as described in our previous published work [19]. Briefly, human hepatocellular carcinoma cells (GS5 cells) were cultured in Dulbecco’s modified Eagle’s medium (DMEM) supplemented with 1× Pen/Strep, 1× non-essential amino acids and 10% fetal bovine serum, and maintained at 37 °C and 5% CO_2_. Cells maintained at 80% confluency were used for all our experiments in this study.

##### C. Cell Viability Assay

We evaluated the biocompatibility of our PEGDA and PEGDA-PCL scaffolds using GS5 cells. We used alamarBlue^®^ to determine the GS5 cell viability on our scaffold at 7, 14 and 21 days using the protocol described in our previous work [20]. All samples were tested in triplicate, and PEGDA scaffolds soaked in media without cells served as a control for our experiments. Each group of scaffolds was sterilized using ultraviolet for 30 min. GS5 cells at a concentration of 1 × 10^5^ cells per 50 µL were seeded onto the scaffold and incubated for 1 h. The scaffolds were then transferred to 12-well plates and cultured in DMEM medium as described above. The plates were incubated for 7, 14 and 21 days and cell culture media was replaced every three days. After an incubation period (7 or 14 or 21 days) alamarBlue^®^ was added at a concentration of 100 µL per mL and incubated for additional 8 h. Contents of each well were mixed using a pipettor, 200 µL were transferred to a 96-well plate and absorbance was determined by a spectrophotometer at 570 nm using 600 nm as a reference wavelength. The total number of viable cells attached to the scaffold was calculated using a standard curve, which was generated by aliquoting cells into a 96-well plate within the range of 50,000–2,000,000 cells/well. A standard curve was generated by plotting number of cells versus absorbance.

##### D. Microscopy and Staining

Attaching GS5 cells to PCL nanofiber, their ability to migrate into a PEGDA scaffold was determined using light microscopy, Haemotoxylin and Eosin (H&E) staining techniques, and confocal microscopy. After 7 and 14 days of incubation, the control and test scaffolds were directly imaged using a Leica (Wetzlar, Germany) light microscope with imaging software at 200× total magnification. For H&E staining, scaffolds were transferred into fresh 12-well plates and rinsed twice with 4.5 mL of PBS to get rid of any nutrient media. Scaffolds were then fixed in 4.5 mL of formalin solution for 4 h at room temperature. After incubation, scaffolds were rinsed twice with 70% ethanol and left in ethanol for 30 min. Then, they were dehydrated using 80, 90 and 100% ethanol with 30 min incubation at room temperature. Scaffold samples were paraffin embedded, sectioned (10–15 µm) and stained by Precision Histology Labs Inc. (Oklahoma City, OK, USA). Stained slides were examined and imaged using Leica light microscope at 400× total magnification.

Attaching GS5 cells to PCL nanofiber and their ability to migrate into PEGDA scaffold was determined using confocal microscopy. After 14 days of incubation, the control and test scaffolds were directly imaged using a Leica SP8 Confocal microscope with LAS X imaging software. For confocal staining, scaffolds were transferred into fresh 12-well plates and rinsed thrice with 4.5 mL of PBS to get rid of any nutrient media. Scaffolds were then fixed in 4.5 mL of formalin solution for 10 min at 37 °C. After incubation, scaffolds were rinsed thrice with PBS and cell nuclei were stained with DAPI (4′,6-diamidino-2-phenylindole) stain for 30 min at 37 °C followed by three washes in PBS.

### 2.4. Statistical Analysis

A one-factor analysis of variance (ANOVA) with subsampling assuming unequal variances was performed using the statistical tools of Kaleida Graph software to determine if there was any significant effect of the application of PCL ENF coating on the mechanical and biological functions of PEGDA. For all statistical tests, *p* < 0.05 was considered as the statistical significant comparison.

## 3. Results

### 3.1. Fabrication of an Electrospinning Unit for the Production of PEGDA Scaffold

**Figure 2:**
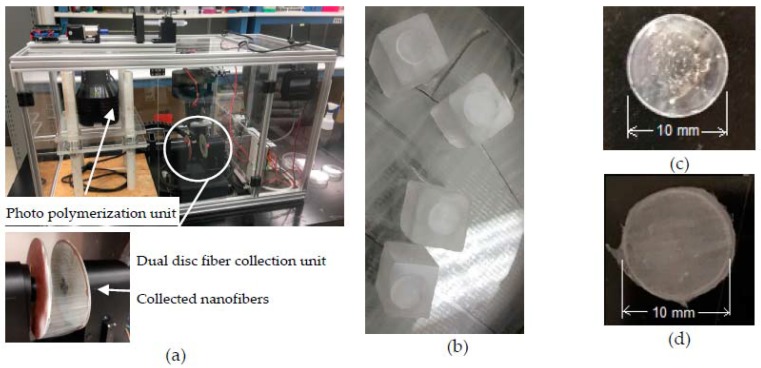
(**a**) an integrated electrospun-UV photo polymerization-machine; (**b**) twelve layers of aligned PCL electrospun nanofiber deposited on the acrylic mold; fabricated samples; (**c**) PEGDA and (**d**) PEGDA-PCL. The thickness of each scaffolds is 1.5 mm.

To conduct this study, the researchers have designed and fabricated an integrated electrospun-UV photo polymerization-machine (Figure 2a) to produce PEGDA-PCL scaffold. The machine utilizes a patented dual disk based mechanism to collect the nanofiber [21]. A robotic arm was developed to automate the machine and reduce the human interference with the machine. The machine is capable of producing twelve layers of aligned uni-direction fibers on an acrylic mold (Figure 2b). The UV photo polymerization unit is used to produce the 1.5 mm thickness and 10 mm diameter PEGDA scaffold (Figure 2c). Both electrospun and UV photo polymerization units are simultaneously used to produce PEGDA-PCL scaffolds where the bottom and top surface of the PEGDA are covered by twelve layers of aligned PCL ENF (Figure 2d). Non-uniform surface topography and voids were observed on PEGDA samples (Figure 2c), where PEGDA-PCL samples were characterized by uniform surface architecture (Figure 2d) due to the coating of PCL ENF.

### 3.2. Surface Characterization

**Figure 3:**
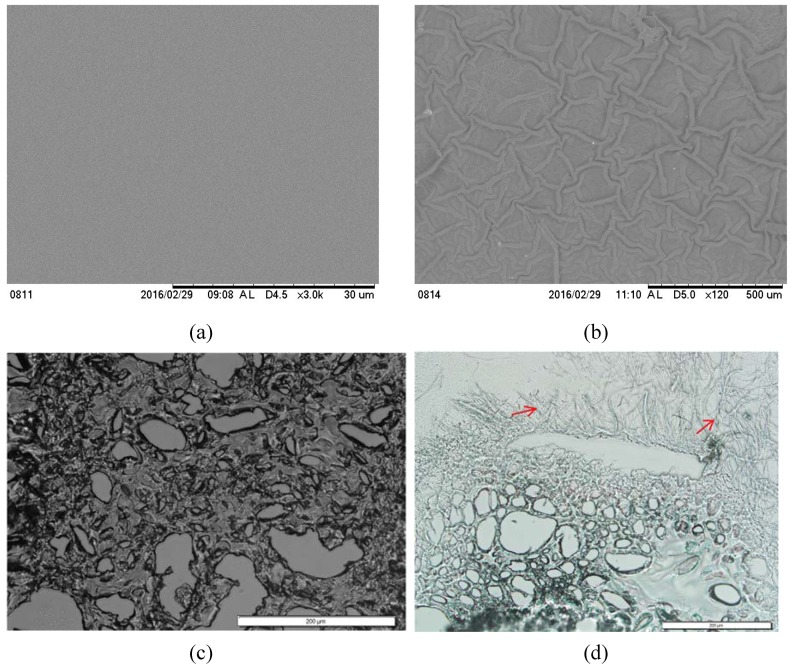
SEM images of the top surface of (**a**) PEGDA and (**b**) PEGDA-PCL scaffolds. PEGDA-PCL samples show a higher amount of artifacts compared to PEGDA. SEM images of paraffin embedded and sectioned scaffolds: (**c**) PEGDA and (**d**) PEGDA-PCL. Red arrows in (**d**) show the presence of PCL ENF in PEGDA. The lengths of the scales bar in (**c**, **d**) are 200 micrometers.

We have compared the SEM images of a fiber layered surface and sectioned image of the PEGDA and PEGDA-PCL scaffold. Higher surface artifacts of PEGDA-PCL scaffold due to PCL ENF arrangement (Figure 3a) were observed when compared to PEGDA scaffold (Figure 3b). The sectioned images of paraffin-embedded PEGDA (Figure 3c) and PEGDA-PCL (Figure 3d) scaffold show the porosity of both scaffolds. The engrossed PCL ENF layers in PEGDA scaffold can be seen from the PEGDA-PCL scaffold.

### 3.3. Mechanical Tests

**Figure 4:**
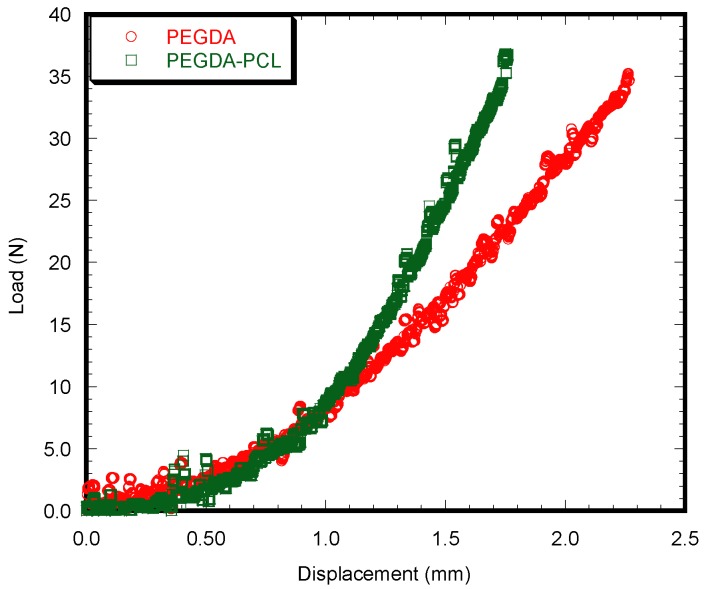
The difference of load vs. displacement behavior between a PEGDA and PEGDA-PCL scaffold during the static compression tests.

There is a significant difference in mechanical properties between PEGDA and PEGDA-PCL scaffolds during the unconfined compression tests (*p* < 0.05) (Table 1). PEGDA-PCL composite scaffold can absorb a higher amount of compressive stress compared to PEGDA under a loaded condition (>10 N) (Figure 4). The average compressive stiffness and modulus of PEGDA-PCL scaffold were higher than that of PEGDA. The results confirmed that PCL ENF membrane could reinforce the PEGDA scaffold.

**Figure 5:**
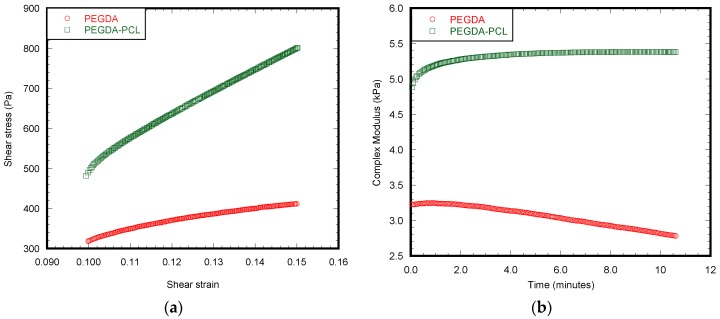
Rheological tests were performed on the hydrogel using the Malvern CVO-100 rheometer at 5% strain rate at frequency 1 Hz. (**a**) shear stress vs. strain and (**b**) complex modulus with respect to time were found from the experiment.

This study reported a significant difference of viscoelastic behavior between PEGDA and PEGDA-PCL scaffolds during the rheological tests (*p* < 0.05) (Table 2). Like the compression test, the rheological tests show that the PEGDA-PCL composite scaffold can absorb a higher amount of shear stress compared to PEGDA due to the increase of shear strain (Figure 5a). The average resultant shear modulus (referred to as complex modulus) of PEGDA-PCL scaffold was higher than that of PEGDA (Figure 5b). The enormous difference of phase angle between PEGDA and PEGDA-PCL confirms that PCL ENF membrane has a strong influence on the viscoelastic characteristics of PEGDA. This happens because the attachment of PCL fiber layers with PEGDA increases elastic modulus, but decreases the viscous modulus.

### 3.4. Bioactivity

**Figure 6:**
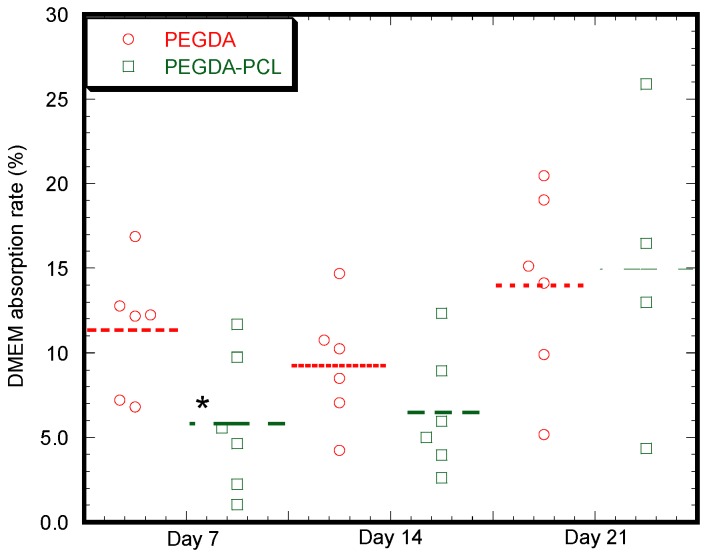
Dulbecco’s Modified Eagle’s medium (DMEM) absorption rate with respect time for PEGDA and PEGDA-PCL scaffolds. Note: * *p* < 0.05 (compared to PEGDA).

Bioactivity of the scaffolds was assessed by soaking both PEGDA and PEGDA-PCL scaffolds in DMEM medium for 7, 14 and 21 days. Both PEGDA and PEGDA-PCL samples show the capability of absorption of DMEM with time (Figure 6). Though the rate of absorption of DMEM for PEGDA samples were found higher for the first week compared to PEGDA-PCL (*p* < 0.5), there is no significant difference in absorption rate after the second and third weeks between the samples. This means PCL fibers do not have any effect on the absorption of DMEM after one week of soaking the sample in the media.

**Figure 7:**
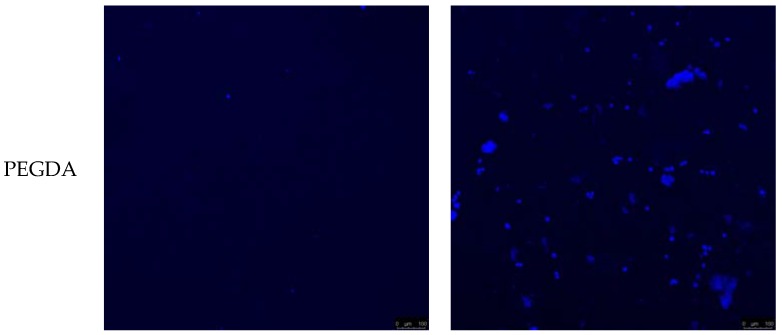

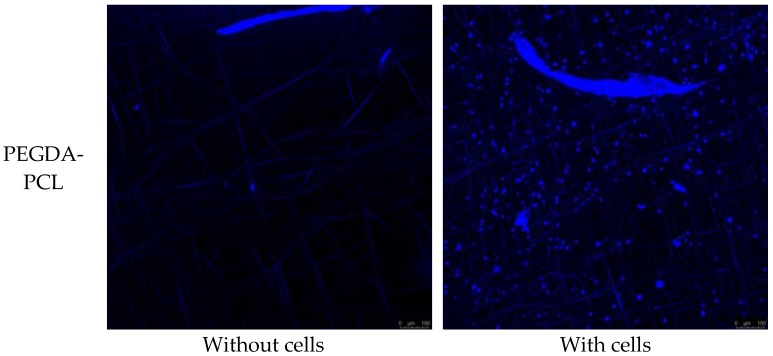
A confocal microscopy image from the top to the bottom surface of PEGDA and PEGDA-PCL scaffold with and without cells after 14 days of cell culture. The blue dots represent cells embedded in PEGDA and PEGDA-PCL scaffolds with DAPI (4’,6-diamidino-2-phenylindole) stained nucleus. The length of the scale bar in each image is 100 microns.

Figure 7 shows the difference of cell migration between PEGDA and PEGDA-PCL scaffolds with and without cells. It is evident from the images that there are more cells in PEGDA-PCL scaffold compared to PEGDA. It also confirms the presence of more cells in the PEGDA-PCL scaffolds compared to PEGDA scaffolds. The confocal images represent a depth of 775 µm from the surface of the scaffolds at 10× magnification.

**Figure 8:**
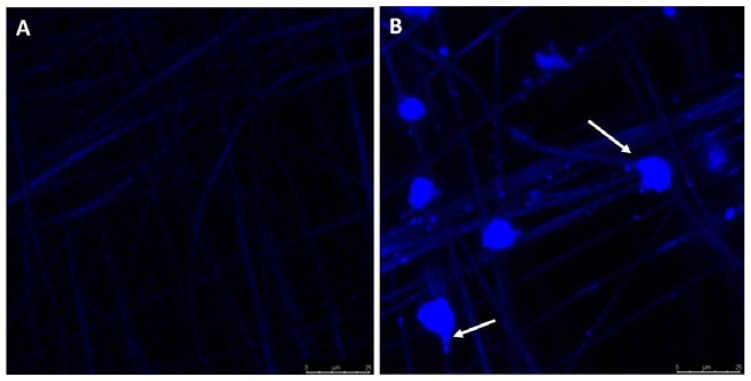
Cell attachment with fiber in PEGDA-PCL scaffold (**B**) after 14 days of cell culture. White arrows point to cells attached to fiber. Panel (**A**) represents PEGDA-PCL without cells. The length of the scale bar in the image is 25 microns.

Higher magnification confocal microscopy images confirm (Figure 8) that the spheroid like cells are attaching onto PCL material using cytoplasmic extensions and more attachment can be seen at the PCL junctions. We have observed a similar trend of increased attachment of osteoblast cells at the PCL junctions on glass slides during the study of the effect of the number of PCL fiber layers on cell adhesion and proliferation on PCL membranes [22].

**Figure 9:**
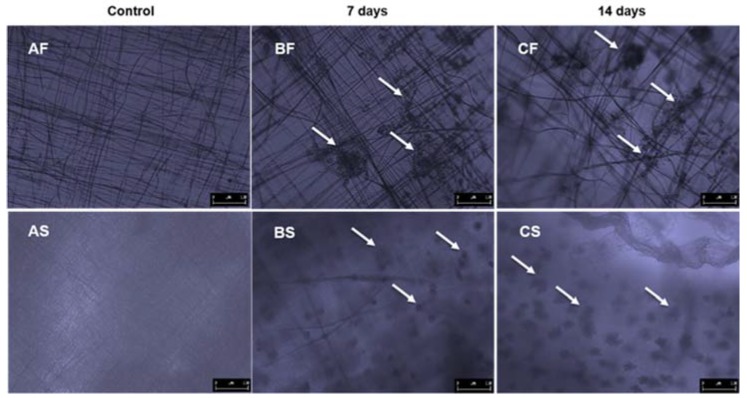
Liver-derived hepatoma cells attached to 12 layers of PCL nanofiber (**BF** and **CF**) sandwiching the PEGDA scaffold; (**BS** and **CS)** represent cells growing embedded in PEGDA; (**AF** and **AS**) are control scaffolds without cells. White arrows point to cells. All scaffolds were incubated for 7 and 14 days. Images were taken using a Leica light microscope at 100× total magnification. The length of the scale bar in each image is 100 microns.

Liver-derived hepatoma cells (GS5 cells) were used to test cytocompatibility of our composite scaffolds after 7 and 14 days of incubation. Figure 9BF shows a light microscope image of hepatocytes attached to PCL nanofiber. The cells are holding onto PCL material using cytoplasmic extensions (Figure 9BF and Figure 9CF) and more attachment can be seen at the PCL junctions. These cells still retain their hepatocyte morphology with an average size of 5–10 µm. As the cells proliferate, they form spheroid like structures attached to PCL fibers. Figure 9BS and Figure 9CS show cells that have migrated into PEGDA hydrogel and formed spheroids. Spheroids appear to be progressing into the PEGDA scaffold. Morphology of hepatocytes in our PEGDA-PCL scaffold closely resemble functional spheroids reported elsewhere [6,23] forming multi-cellular aggregates. The increase in spheroid size between 7 and 14 days shows that nutrients and oxygen are able to percolate through the material and support cell growth.

**Figure 10:**
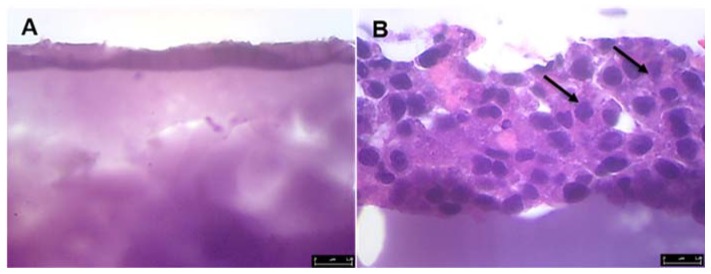
Haemotoxylin and Eosin (H&E) staining of PEGDA-PCL scaffolds with GS5 cells (**B**) after 7 days. Black arrows in (**B**) point to cells; (**A)** is control scaffold without any cells. Leica light microscope at 400× magnification. The length of the scale bar in each image is 100 microns.

Our H&E staining results (Figure 10) also corroborate this observation. It shows that cells have migrated from the top (the area where the fibers were deposited) and moved towards the other side of the scaffold.

**Figure 11:**
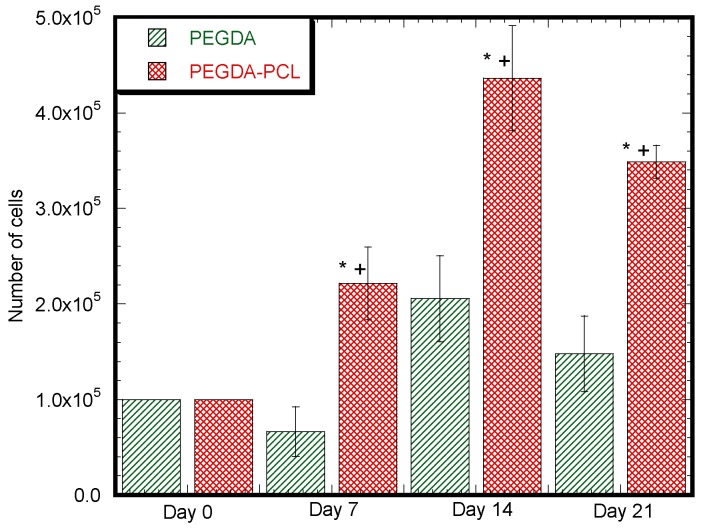
Increase in cell number on PEGDA-PCL scaffold with time. GS5 cell Viability after 7, 14 and 21 days on PEGDA and PEGDA-PCL scaffolds using alamarBlue^®^ assay. Our results indicate that cells remain viable in our scaffolds. Values are mean ± SOE of triplicates. In the figure, * *p* < 0.05 (compared to Day 0) and + *p* < 0.05 (compared to PEGDA for the same day).

Cell viability was measured using alamarBlue^®^ with results shown in Figure 11. Our results clearly indicate that the viability of cells in PEGDA-PCL at 7, 14 and 21 days was significantly higher than PEGDA samples (*p* < 0.05). Cells are proliferating in PEGDA samples after 14 days. There is a decrease in the amount of proliferation after 21 days in comparison to 14 days. During the three week of incubation of PEGDA-PCL scaffolds, there is a significant increase in cell number (*p* < 0.05) and the cells continued to proliferate in all PEGDA-PCL samples. On our PEGDA samples, there is also a decrease in cell proliferation after 21 days compared to 14 days.

## 4. Discussion

This study has successfully fabricated electrospun-UV polymerization systems that can produce 10 mm diameter and 1.5 mm thickness PEGDA-PCL scaffold. This machine is capable of assembling PCL and PEGDA membranes simultaneously in layers to produce larger thickness and custom shape PEGDA-PCL scaffolds. Such scaffolds can meet the mechanical and biological requirements for successful integration with the human body, which is the future direction of this research.

We have observed an increased amount of DMEM absorption capabilities with time for both PEGDA and PEGDA-PCL samples due to the porosity of both samples. There is a statistical difference on day 7 (*p* < 0.05) and a noticeable difference on day 14 between PEGDA and PEGDA-PCL scaffolds (Figure 6). The volume of the surface and internal voids expand with time due to degradation, which is higher for PEGDA-PCL scaffolds compared to PEGDA due to the presence of PCL. The amount of void increases with time, which may contribute to the higher amount of degradation and DMEM absorption rate for PEGDA-PCL with time. Compared to PEGDA, Figure 6 shows reduced absorption of DMEM by PEGDA-PCL at 7 and 14 days, but, at Day 21, no difference of absorption is noticed between PEGDA and PEGDA-PCL scaffolds. Thus, PEGDA performs better in providing nutrients for the cells at least during the first two weeks. Although the study shows that a lower amount of cells adhered to PEGDA surface compared to PEGDA-PCL due to a reduced amount of surface artifacts; therefore, the increasing amount of nutrients in PEGDA did not contribute to the higher amount of cell growth and proliferation compared to PEGDA-PCL (Figure 11).

Results showed that our developed scaffolds satisfied the minimum compressive modulus requirement for bone graft substitutes (>0.5 MPa [24]). Further improvement of stiffness and other mechanical properties of PEGDA scaffold is possible by optimizing the porosity of PCL ENF membrane and immobilization of osteoconductive nanoparticles such as hydroxyapatite, MgO with PCL nanofiber in the scaffold. The other way is to increase the number of layers of PCL and PEGDA membranes. PCL membranes provide interconnectivity between two PEGDA membrane layers. They can serve as an entrapment device to provide the required structural strength, nutrient transport, and micro-environment for cell and tissue in-growth to the adjacent PEGDA layers.

A 3D scaffold provides a 3D environment in which cells are better able to mimic their in vivo counterparts. Our results clearly demonstrate that twelve layers of PCL nanofiber on 1.5 mm think PEGDA provides an ideal surface area for cell attachment, allowing transfer of nutrients, oxygen and removal of metabolic waste from cell growth sites. PCL nanofiber provides an ideal surface for cells to spread well and attach firmly (Figure 9). Considering the results from cell viability and staining (Figure 10 and Figure 11), we are convinced that our composite scaffold is ideal for cell response and adhesion.

The variability of the physical and mechanical characteristics in this study was due to the difference of the ENF and PEGDA membrane thickness and architectures in PEGDA-PCL scaffolds. It is impossible to collect the same ENF architecture from the harvested aligned uni-direction ENFs using the dual disc collector. There is variability in hydrogel membrane thickness and architectures in PEGDA and PEGDA-PCL scaffolds since it is impossible to deposit the same amount of hydrogel and cure under the same conditions using the photo-polymerization curing systems. These limitations can be overcome by fine-tuning the integrated UV photo-polymerization system as well as testing a large number of samples.

This study is related to increasing the bio-physio-mechanical properties of hydrogel using ENF membranes, particularly PEGDA hydrogel. A literature search has revealed no reported research directed to PEGDA-PCL scaffold in relation to the influence of PCL ENF coating on the physical, mechanical and biological performances of PEGDA-PCL scaffold. Therefore, we are unable to verify our results with others.

**Figure 12:**
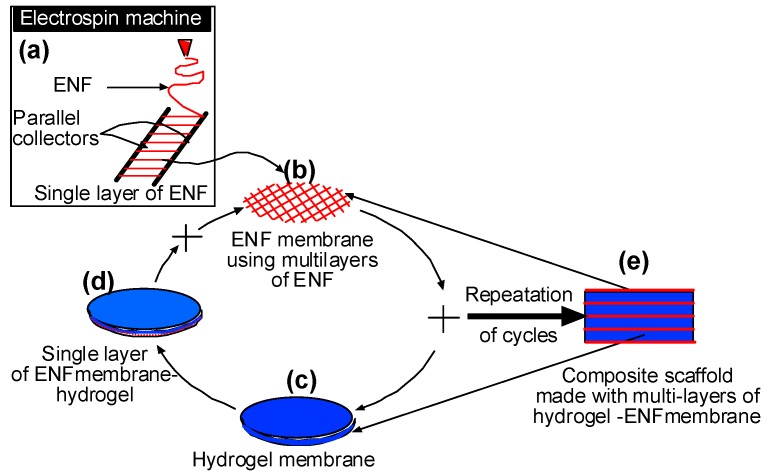
Schematic to produce 3D scaffold using electrospun nanofiber (ENF) membranes.

The study revealed that PEGDA-PCL scaffolds provide a fundamental aspect for producing a tissue scaffold with better mechanical and biological capabilities than a scaffold comprised with PEGDA only. Studies investigating the influence PCL ENF has on PEGDA-PCL scaffolds are required for the design of PEGDA- PCL scaffolds that produce better clinical outcomes for tissue engineering applications. In the authors’ research, the PEGDA and PCL membranes are stacked and interspersed between multiple layers of PCL membranes, binding them together to produce a three-dimensional (3D) composite scaffold as schematically presented in Figure 12. This patent pending approach of creating composite scaffolds made with multilayers of PCL ENF and PEGDA hydrogel membranes is unique [15]. This process of producing 3D scaffolds can be used for various tissue engineering applications. The authors’ research group is currently developing 3D scaffolds for skin equivalent models [7], an in vitro cell culture test system to understand the mechanics of liver cells [19], and nucleus pulposus scaffolds for degenerated discs [25]. PEGDA-PCL based scaffolds for tissue engineering applications has been reported by other researchers. For example, Zhao et al. [26] developed bio-artificial blood vessels by coating PCL using PEGDA. Correia et al. [27] developed UV cross-linked gelatin coated electrospun PCL fibrous scaffolds for assessing the chemical and physical properties as well as their interaction with blood and endothelial cells for cardiovascular disease. Morris et al. [28] fabricated ear-shaped chitosan-PEGDA scaffolds by a stereolithographic method. Shanjani et al. [29] fabricated and characterized well-defined porous PCL- beta-tricalcium phosphate scaffolds with identical pore size (500 µm), but different strut sizes (200 and 400 µm), using additive manufacturing technology and filled the porous scaffold by PEGDA hydrogel. The process of producing PEGDA-PCL scaffolds and the architecture of the produced scaffold is significantly different from the previous researchers. The main purpose of sandwiching PCL membranes between two adjacent layers of PEDGA is that PCL membranes can serve as a reservoir for the supplies of biominerals. In addition, it can be used to control the dispersion of cells homogeneously to the PEGDA layers. The scaffold requires carrying physiological load when implanted in the body. The PCL fiber can reinforce PEGDA for a nucleus pulposus scaffold for a degenerated disc.

The limitation of the study is that this study qualitatively shows the size and distribution of pores in PEGDA and PEGDA-PCL scaffolds from histological and confocal section images, but no quantitative measurement of the size and distribution of pores in scaffolds was conducted. Another limitation of the study is that this study tested only hepatocytes as a cell prototype to compare the biocompatibility of the PEGDA and PEGDA-PCL scaffolds. In previous research, Khandaker et al. [7] used fibroblast cells to show that networked PEGDA hydrogels, where macrosize networked channels were manually created in PEGDA. The study found that networked PEGDA possessed increased cell viability compared to non-networked and decreased cell viability with increased photoinitiator concentrations. This study is the continuation of the previous study where the networked structure in PEGDA was created by a PCL nanofiber matrix. Currently, the authors’ research group is using fibroblast cells in a PEGDA-PCL based skin equivalent model to evaluate the biomechanical performance of an ear tube. In addition, we are performing cytocompatibility analyses of PEGDA-PCL with varying number of layers (12 layers vs. 24 layers) for 7, 14 and 21 days using adult human mesenchymal stem cells. We have used osteoblast cells to understand the effect of architecture of PCL fiber layers on cell viability on PCL membranes [22]. Another limitation of the study is the use of the hepatocellular carcinoma cells in the scaffold instead of using primary liver or skin (fibroblasts) cells. In the authors’ previous work [19], human hepatoma cells were used as a model system to test the application of different scaffold materials for liver tissue engineering. They have used the same cell line for this research, as it will help them compare their current work with previous work. In the authors’ future research, the authors plan to use normal human hepatocytes and adult stem cells to test the feasibility of the current scaffold system.

## 5. Conclusions

This study shows a method for the incremental advancement and translation of PCL ENF to produce a functional PEGDA based scaffold for tissue engineering. The feasibility of depositing multiple layers of PCL ENF on PEGDA was explored. This study shows that the mechanical and biological performances of PEGDA scaffold can be significantly improved by using a highly organized and high porosity electrospun nanofiber matrix. This study will lead to the production of a novel PEGDA-PCL composite scaffold that may have competing performances with the commercially available scaffolds (e.g., 3D cell culture products from Nanofiber Solutions (Hilliard, Ohio, USA) or 3D Biotek, LLC, Hillsborough, New Jersey, USA) for biomedical applications.

## Figures and Tables

**Table 1:** Difference between mechanical properties between PEGDA and PEGDA-PCL scaffolds during compression. Both samples have the same diameters (10 mm) and were loaded up to 35 N to calculate the mechanical properties. Data presented *n* = 3 for both samples. Data presented as a mean ± standard error. Note: * *p* < 0.05 (compared to PEGDA).

Experimental Parameters	PEGDA	PEGDA-PCL
Compressive stiffness (N/mm)	3.00 ± 0.12	5.36 ± 0.02 *
Compressive modulus (kPa)	259.68 ± 3.56	509.61 ± 0.01 *

**Table 2:** Difference between viscoelastic properties between PEGDA and PEGDA-PCL scaffolds. Both samples have the same diameters (9.56 mm). Data presented *n* = 3 for both samples. Data presented as mean ± standard error. Note: * *p* < 0.05 (compared to control).

Experimental Parameters	PEGDA Only	PEGDA-PCL
**Viscous modulus (kPa)**	1.01 ± 0.24	0.53 ± 0.18 *
**Elastic modulus (kPa)**	2.82 ± 0.12	5.34 ± 0.23 *
**Phase shift angle (degree)**	19.72 ± 0.40	5.68 ± 0.22 *
